# Alignment-free detection of differences between sequencing datasets

**DOI:** 10.1016/j.isci.2025.113828

**Published:** 2025-10-24

**Authors:** Alessia Petescia, Luca Denti, Askar Gafurov, Viktória Hodorová, Jozef Nosek, Broňa Brejová, Tomáš Vinař

**Affiliations:** 1 Department of Applied Informatics, Faculty of Mathematics, Physics and Informatics, Comenius University in Bratislava, Bratislava, Slovakia; 2 Department of Computer Science, Faculty of Mathematics, Physics and Informatics, Comenius University in Bratislava, Bratislava, Slovakia; 3 LIRMM, University of Montpellier, Montpellier, France; 4 Department of Biochemistry, Faculty of Natural Sciences, Comenius University in Bratislava, Bratislava, Slovakia

**Keywords:** Techniques in genetics, Biocomputational method, Genomic analysis, Sequence analysis

## Abstract

Comparing biological samples through sequencing is a core task in bioinformatics analyses such as variant detection, differential expression analysis, and epigenetic peak calling. Standard approaches typically rely on mapping newly sequenced reads to a reference genome. To avoid mapping and reference biases, *k*-*mer*-based approaches have been proposed as an alternative. Using this paradigm, our tool kdiff identifies genomic regions containing *k*-mers with differential abundances between samples. We demonstrate that our method effectively detects copy number variants in cancer genomes and remains robust against reference genome misassemblies. Additionally, we illustrate its utility in confirming telomere locations in noisy nanopore sequencing data. Our work demonstrates that alignment-free approaches can provide results comparable to standard alignment-based methods, while reducing the reference bias and significantly improving computational efficiency by leveraging fast *k*-mer counting tools.

## Highlights

• Alignment-free method kdiff finds differences between unassembled sequenced samples
• It is robust to low-quality references and low-quality/low-coverage sequencing data
• Kdiff is significantly faster than alignment-based methods

## Graphical abstract

**Figure:**
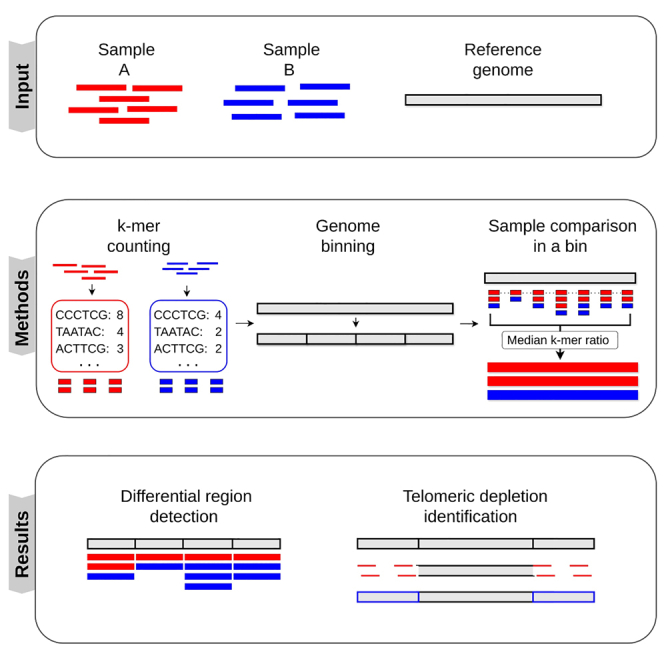

## Introduction

Understanding genetic variation is fundamental to deciphering biological diversity, disease mechanisms, and phenotypic traits across species.^1^^,^^2^^,^^3^^,^^4^^,^^5^ With many cost-effective DNA sequencing technologies available, genomic sequencing has become a tool of choice for comparing biological samples. Many approaches have been developed to identify genomic differences between two sets of sequencing reads, with alignment-based methods being the most widely used. These techniques map sequencing reads to a reference genome and analyze read depth variations or unexpected alignments, such as split reads or anomalous insert sizes.^6^^,^^7^^,^^8^^,^^9^^,^^10^^,^^11^^,^^12^ While generally effective, biases related to GC content, mappability, and sequencing errors can confound results. Additionally, reference genomes must be complete and accurate, as missing sequences, misassemblies, and sequence divergence can introduce errors, making it difficult to detect variations in highly repetitive or structurally complex regions.^13^ Mapping biases arise because some genomic regions, particularly repetitive sequences, do not uniquely map to a single location, causing misinterpretations of read depth and improper variant calls.^7^ Furthermore, sequencing reads that diverge significantly from the reference genome may be discarded or misaligned, leading to an underestimation of genomic differences.^14^ The computational cost of alignment-based approaches is also substantial, especially at high sequencing depths, further motivating the development of alignment-free methods.

Alignment-free approaches^15^^,^^16^^,^^17^^,^^18^^,^^19^^,^^20^^,^^21^^,^^22^^,^^23^ usually compare sequencing read datasets by analyzing their *k-mers* (substrings of length *k*) within a sequence. These methods generally fall into two categories: those based on *k*-mer presence/absence and those based on *k*-mer abundances. Presence/absence-based methods operate under the assumption that novel genetic variations introduce new *k*-mers that were not present in the reference genome, if *k* is sufficiently large.^15^^,^^16^ By identifying these unique *k*-mers, these methods can detect insertions of novel sequences and breakpoints introduced by deletions and other structural variants.

Abundance-based approaches rely on the principle that variations affecting copy number, including insertions, duplications or deletions, will lead to changes in *k*-mer frequencies.^18^^,^^19^^,^^20^^,^^21^^,^^22^^,^^23^ Among existing approaches, the methods proposed by Rahman et al.^18^ and Wang et al.^19^ are among the most widely adopted and exhibit the greatest similarity to our own, which will be described in subsequent sections. These methods operate by comparing two sets of sequencing reads through statistical analysis of *k*-mer frequency distributions. By examining differences in *k*-mer abundance across samples, they identify *differential k-mers*, i.e., *k*-mers whose frequencies differ significantly between conditions. Subsequent steps typically involve assembling these differential *k*-mers directly, or assembling reads that span them, to reconstruct longer variant sequences. As a result, the output generally consists of either a list of differential *k*-mers or a collection of assembled sequences. However, to interpret the biological significance of these sequences and classify the type of genomic variation they represent, it is often necessary to map them back to a reference genome, if one is available. This step can reintroduce some of the biases that alignment-free methods are designed to avoid, particularly in repetitive regions such as those affected by copy number variations (CNVs). Furthermore, when mapped back to the genome, CNVs are typically represented as insertions or deletions rather than being directly quantified, complicating the inference of absolute copy number. In spite of this drawback, these approaches have been widely applied in genome-wide association studies (GWASs),^24^^,^^25^^,^^26^^,^^27^^,^^28^ in some cases achieving better results when compared to classical methods.^26^ However, identification of sequence variation events underlying significant differential *k*-mers remains a challenge.^25^^,^^26^

In this work, we present kdiff, a novel *k*-*mer*-based approach for differential analysis that integrates alignment-free principles with reference genome information. It requires as input two sets of sequencing reads to be compared and a reference genome. Instead of relying on mapping, kdiff partitions the reference genome into non-overlapping windows of user-defined length and determines the degree of enrichment or depletion of each window based on its *k*-mer abundance profiles. To achieve this, *k*-mer counting is first performed separately for each set of sequencing reads. The absolute *k*-mer counts are then normalized to account for differences in total sequencing depth between samples. Finally, median of relative *k*-mer abundances are reported for each window of the reference genome. The use of median smooths out outliers caused by single nucleotide polymorphisms, homopolymers, high-frequency *k*-mers and other phenomena. By leveraging the ratio of relative *k*-mer abundances between two samples, kdiff inherently accounts for sequencing biases. Assuming both samples are sequenced using the same technology, systematic biases affecting *k*-mer counts will cancel out and do not need to be handled explicitly, greatly simplifying the method and making it directly applicable to diverse sequencing technologies.

While an early version of our method^29^ was evaluated only on a single toy simulated dataset, in this paper, we demonstrate kdiff functionality on two biologically relevant case studies. First, we assess its effectiveness in detecting CNVs in cancer samples by comparing sequencing data from healthy and cancerous tissues. We benchmark our results against those obtained using CNVkit,^6^ a widely used CNV detection tool, demonstrating that kdiff achieves comparable accuracy while offering improved computational efficiency and robustness to reference genome misassemblies. Next, we explore the applicability of kdiff in analyzing low-coverage nanopore sequencing data from yeast, focusing on telomeric regions. These regions are particularly challenging due to their repetitive nature and the absence of a highly accurate reference genome for the specific yeast strain studied. We demonstrate kdiff successfully detects biologically meaningful depletion patterns at chromosomal ends, highlighting its suitability for studying complex genomic regions and non-model organisms in cases where a reliable reference genome is unavailable. Our results showcase the versatility of kdiff across different sequencing technologies and experimental conditions.

## Results

### Copy number variation in cancer samples

In this section, we apply kdiff to the task of detecting CNV by comparing samples from healthy and cancer tissues. Specifically, we used whole-genome sequencing reads obtained with NovaSeq technology (151 bp read length), with estimated sequencing coverages of 73x for normal tissue and 80x for cancer tissue with 100% purity level produced by the SEQC2 Consortium.^30^ Both samples originated from the same patient, a woman diagnosed with triple-negative breast cancer (TNBC). The normal tissue was derived from a B lymphocyte cell line (HCC1395BL), while the tumor sample came from a corresponding cancer cell line (HCC1395).^31^

We compare our results to CNVkit,^6^ a widely adopted tool that employs a mapping-based approach optimized for short-read sequencing data. CNVkit divides the reference genome into windows of user-specified mean length. To facilitate direct comparison, we modified kdiff to use the same window boundaries as CNVkit. All analyses were performed using windows with a mean length of 1 kb and *k* = 21 for *k*-mer counting, as recommended by Philippe et al.^32^ for human genome analysis.

### Kdiff and CNVkit produce highly correlated results for hg38 reference assembly

**Figure 1:**
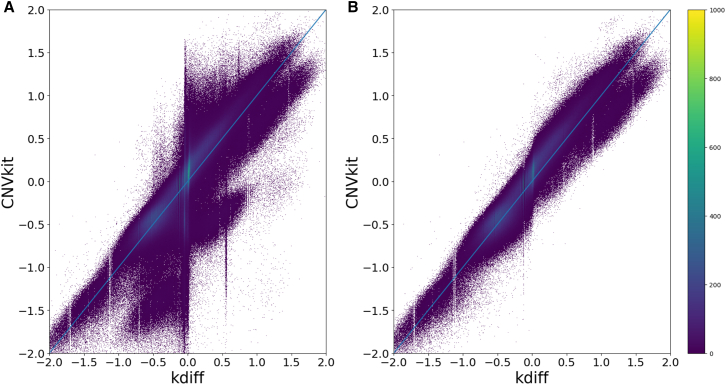
Comparison of kdiff and CNVkit results on the hg38 assembly Each point in the scatterplot represents log2 ratios assigned to a single window by the two tools. The color represents density of the points. (A) Unfiltered results (correlation 0.78). (B) Results after removing windows from chromosome X and Y and those affected by repeats (correlation 0.87).

When applied to CNV detection in cancer tissues, kdiff and CNVkit produce highly correlated results (correlation coefficient 0.78); however Figure 1A also highlights certain discrepancies. In particular, two distinct clusters of windows displayed systematic differences, with kdiff consistently predicting values one copy higher or lower than CNVkit. Upon further investigation, we determined that these windows were located on the sex chromosomes (X and Y). This discrepancy arises because CNVkit automatically infers the sample’s sex from the BAM file, adjusting the normalization factors for these chromosomes accordingly. In this case, CNVkit mistakenly classified the sample as male, leading to incorrect normalization.

Second, we observed that for some windows kdiff assigned a *log*_2_ ratio of 0 (neutral copy number of 2), whereas CNVkit assigned various non-zero ratios. These windows corresponded to tandem repeat regions. This discrepancy is rooted in the fundamental differences between the two methods. CNVkit relies on read mapping and in some cases single nucleotide polymorphisms distinguish repeat copies and help to uniquely assign reads to a specific location. kdiff, on the other hand, does not rely on mapping and instead considers only *k*-mers present in the reference genome. Consequently, it assigns the same ratio to identical *k*-mers across the genome, effectively reporting the average copy number of a given sequence rather than resolving copy number differences within repeat regions. While this can be a limitation in cases where a small copy number change occurs within a large repeat cluster, this behavior can be advantageous in other scenarios, especially when the reference genome quality is lower.

These two factors explain majority of significant differences between kdiff and CNVkit results, as shown in Figure 1B which excludes windows from chromosomes X and Y and windows affected by repeats. These were defined as windows with median *k*-mer count in the reference above 1.

### Kdiff is significantly faster than CNVkit

We measured the running time of various tasks required for analysis of the above datasets with kdiff and CNVkit. Table 1 shows that the major bottleneck of analysis with CNVkit is the alignment of the sequencing reads to the reference genome, which has taken more than 18 h in our experiments. In comparison, the *k*-mer counting in kdiff analysis has taken less than 2.5 h for our datasets.

**Table 1:** Comparison of the running times of various analysis steps for kdiff and CNVkit

Tool	Process	Time	Space	Threads
		(h:m:s)	(GB)	
**CNVkit**	bwa index (hg38)	53:48	4.7	//
	bwa mem (control)	8:14:11	15.2	24
	bwa mem (case)	9:24:38	15.4	24
	CNVkit	55:05	3.9	24
Kdiff	kmc count (hg38)	1:31	14.5	24
	kmc count (control)	1:04:21	15.8	24
	kmc count (case)	1:13:34	15.8	24
	kmc filter (control)	8:03	11.4	//
	kmc filter (case)	7:57	11.4	//
	kdiff	39:46	18.6	//

The table reports the execution times, memory usage, and the number of threads used for each process. All experiments were measured using the time function, with hg38 as the reference genome and *k* = 21. *k*-mer counting was performed using KMC3, while read mapping was carried out using bwa mem.

### Misassemblies lead to artifacts in reference-based tool predictions

We have used simulated data to assess whether kdiff could accurately detect CNVs occurring in regions of the reference genome affected by misassemblies and to compare its performance with CNVkit. We simulated four types of misassemblies: *(A)* insertion, *(B)* deletion, *(C)* repeat collapse, and *(D)* repeat expansion. Insertions and deletions correspond to the erroneous addition or loss of sequences in the reference genome, whereas repeat collapses and expansions affect multi-copy repeats. In these cases, assemblers may mistakenly merge different repeat copies or introduce extra copies of a repeat during the assembly process, leading to misassemblies.^33^^,^^34^

**Figure 2:**
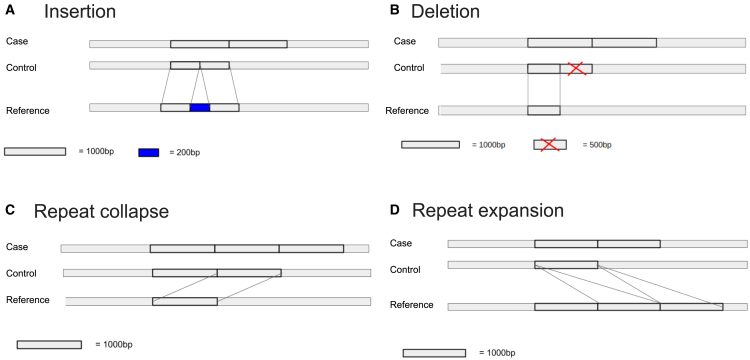
Overview of simulated experiments For each experiment, we start with a randomly generated control genome. The case genome is created by duplicating a 1000 bp region. A separate reference genome represents a missassembled version of the control genome. (A) The reference contains an additional 200 bp novel insertion in the middle of the region affected by duplication. (B) Half of the affected region (500 bp) is missing in the reference. (C) While the control genome contains two copies of the affected region, and the case genome contains three copies, the reference only contains a single copy. (D) The reference genome contains three copies of the affected region, compared to one in the control genome and two in the case genome.

For each experiment, we start with a randomly generated control genome. The case genome is created by duplicating a 1000 bp region of the control genome. A separate reference genome represents a misassembled version of the control genome, where the misassembly affects the region with the duplication (Figure 2). Next, for each control and case genome, sequencing reads of length 151 bp were simulated using Insilicoseq^35^ with the NovaSeq error model, applying a coverage of 15x for the case genome and 20x for the control genome. Thus for each of the four experiments, we have necessary data consisting of one reference genome, one case read set and one control read set.

In each of the experiments, we ran kdiff to compare the case and control read sets using the respective reference genome. We also aligned the read sets to the reference using bwa-mem^36^ in order to apply CNVkit. Analyses were repeated using window sizes of 300, 500, and 1,000 bases, with *k* = 21. Each window was subsequently classified as *absent* if the reported abundance ratio was smaller than 0.45, *depleted* if it was between 0.45 and 0.75, *neutral* if it was between 0.75 and 1.45, and *enriched* if it was greater than 1.45.

Regardless of the chosen window size, both kdiff and CNVkit successfully identified the gain in all cases, except for experiment D (repeat expansion). In this case, CNVkit classified the windows spanning the beginning of the first repeated region and the end of the last repeated region as normal, while excluding all the windows in between from its predictions. In contrast, kdiff correctly identified all repeated regions as a gain. This discrepancy can be attributed to CNVkit’s reliance on its read-depth calculation, which excludes secondary alignments and filters out windows where the read depth of the control sample falls below a predefined threshold. Read depth in repeated regions is inherently influenced by the behavior of standard mapping tools. When a sequencing read aligns equally well to multiple locations, the mapper randomly assigns it to one, attempting to distribute reads evenly. If the reference genome contains more copies of a particular segment compared to the case/control samples, read counts can become dispersed, potentially leading to undetected CNVs in these regions.

In real datasets, variation in the number of segmental duplications (also known as low-copy repeats) between samples is common. For example, approximately 5% of the human genome is comprised of segmental duplications ranging from 1 to 400 kb in length and sharing high sequence identity (greater than 90%).^37^ When a *k*-mer appears at multiple locations in the reference genome, it is always assigned the same count and ratio in kdiff. Consequently, kdiff predictions in duplicated regions may not always represent the true state of that particular copy but instead summarize the total copy number change between the samples. This behavior is easy to interpret and is independent of the quality of the underlying reference assembly which may contain various artifacts such as expansions or contractions in such regions. In contrast, while CNVkit under ideal conditions may be able to provide the state of a particular repeat copy, our experiment shows that the results are likely affected by assembly artifacts and details of the read mapping algorithm, which makes it difficult to interpret the results in the end.

### Kdiff shows significantly more robustness of predictions between genome assemblies

In previous section, we have shown that reference assembly quality may impact the results of the CNV predictions. To assess the robustness of CNV predictions with respect to the choice of a reference genome on real human data, we applied kdiff and CNVkit to the aforementioned cancer dataset using three different human genome assemblies: hg17, hg38, and hs1. We then compared the *log*_2_ ratios assigned to corresponding genomic windows across these assemblies. To facilitate this comparison, genomic coordinates were mapped between assemblies using the liftOver tool,^38^ and only windows where at least 90% of the lifted coordinates overlapped with the corresponding window in the target genome were considered.

**Figure 3:**
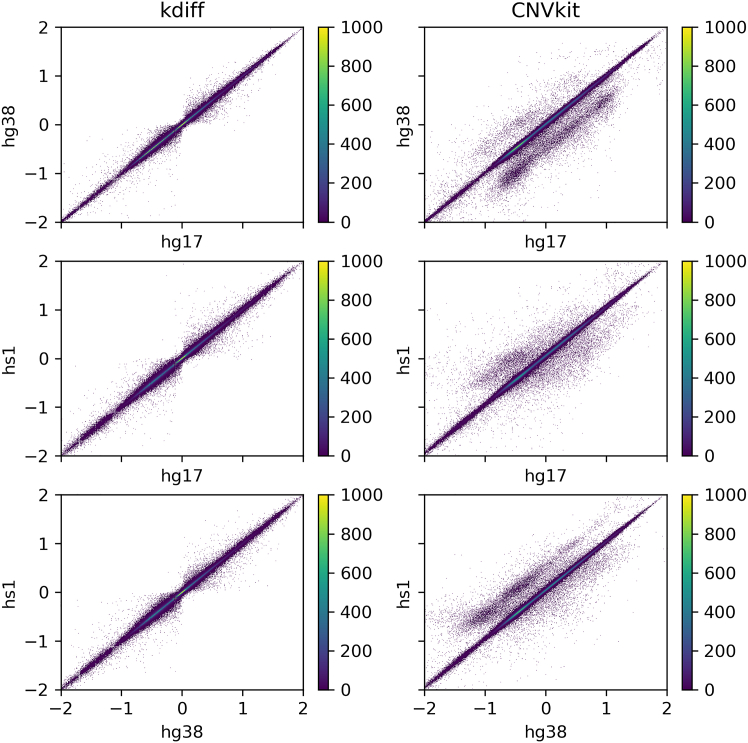
Robustness of kdiff and CNVkit predictions across different genome assemblies (hg17, hg38, hs1) Each point represents a genomic window, with axes showing log_2_ ratios assigned by the same tool using different reference genomes. Since the underlying data remain unchanged, log_2_ ratios should be consistent across assemblies. The results indicate that kdiff provides more stable predictions regardless of the reference genome used.

As illustrated in Figure 3, kdiff produced more consistent predictions across all three assemblies. In contrast, CNVkit exhibited systematic biases when comparing hs1 and hg17 to hg38, as evident from distinct clusters of higher or lower *log*_2_ ratios in certain windows. Upon further investigation, we determined that hg38, unlike hg17 and hs1, includes multiple alternative versions of each chromosome, which contain nearly identical sequences. Since all versions were included in the analysis, the mapping process resulted in reduced read counts in these regions, leading CNVkit to assign lower ratios compared to the assemblies lacking these duplicated regions. Again, this behavior is consistent with what we observed on our artificial data.

Motivated by these observations, we further investigated the impact of segmental duplication differences across assemblies on CNV prediction consistency. To this end, we retrieved the lists of segmental duplications in hg38 and hs1 from the UCSC Genome Browser.^39^ We focused on regions that appear multiple times in hg38 but are present only once in hs1 and we considered matched windows from the previous analysis that are inside these regions. Subsequently, we calculated the mean absolute error (MAE) of the *log*_2_ ratios assigned to the windows by each tool in different assemblies. CNVkit exhibited MAE of 0.08, whereas kdiff achieved a significantly lower MAE of 0.001, demonstrating superior consistency of predictions in the presence of segmental duplications.

### Telomere identification

To further evaluate the applicability of our method, we generated and analyzed a new dataset inspired by the BAL31-NGS protocol for discovery of telomeric sequences.^40^ In this protocol, high-molecular-weight genomic DNA is extracted from cells. A control sample is sequenced unmodified, whereas a second portion is first digested with BAL-31 nuclease.^41^ This enzyme degrades and cleaves at double-stranded DNA from both termini nicks and single-stranded regions. Although such nicks and breaks will occur at random positions in the genome, chromosome ends (natural double-stranded breaks) will be degraded in all chromosomal copies and thus will be underrepresented in sequencing reads compared to the control sample. This situation is similar to CNV loss, but differs in that boundaries of the depleted region are not sharp, but instead we expect a gradient from strong depletion to normal coverage. Note that telomeric and subtelomeric regions are highly repetitive and the repeats are shared among chromosomes. Thus this scenario is particularly well suited to our method, which can pool signal from repeated *k*-mers.

We applied this protocol combined with long-read nanopore sequencing to produce new data from yeast *Lodderomyces elongisporus* strain CBS 5301 (see STAR Methods), resulting in one control and two treated samples obtained with different enzyme concentrations. We refer to treated samples as *low-treated* (b05) and *high-treated* (b06), respectively. As the reference genome, we used the GCF_030384665.1 assembly of a different strain (NRRL-YB4239) of the same yeast species.^42^ We compared each treated sample with the control using kdiff with varying *k*-mer lengths (*k*∈{15,21}) and window sizes (*w*∈{100,300,500,1000}). We then examined the distribution of depleted windows across the genome.

**Figure 4:**
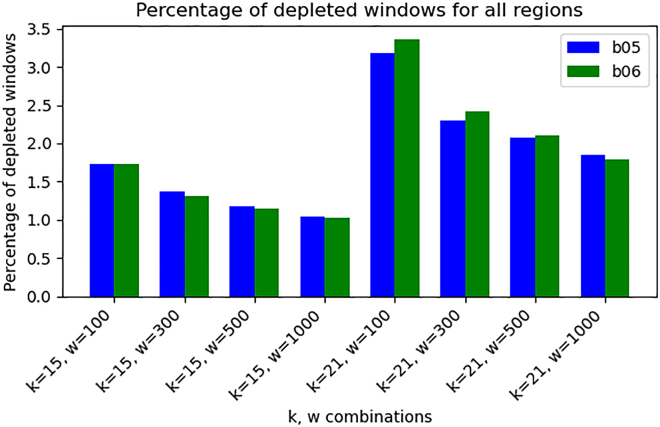
Genome-wide comparison of DNA depletion in yeast samples subjected to different durations of enzymatic degradation Samples b05 (*low-treated*) and b06 (*high-treated*) were exposed to a treatment designed to degrade DNA from both ends of double-stranded molecules. If the treatment was effective, the total number of depleted windows (log_2_ ratio ≤ -1) should remain similar across the whole genome, with differences expected primarily at chromosome ends. The percentage represents the total number of depleted windows in a sample divided by the total number of windows in the sample’s genome. Each pair of bar plots represents results for the two samples using different combinations of *k*-mer length (*k*) and window size (*w*). The plot confirms that the overall proportion of depleted windows is comparable between the two samples.

**Figure 5:**
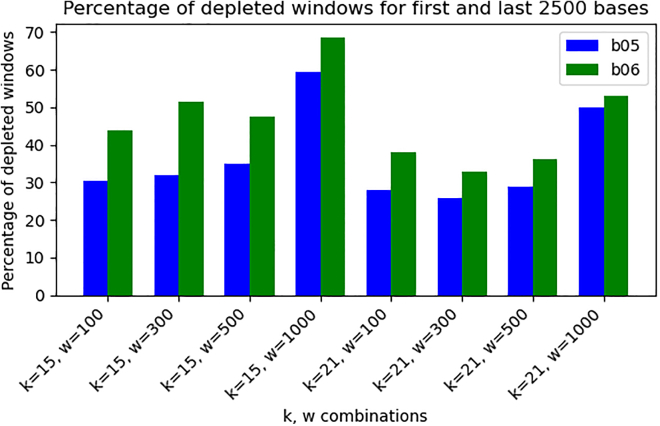
Comparison of DNA depletion at chromosome ends in yeast samples subjected to different durations of enzymatic degradation Samples b05 (*low-treated*) and b06 (*high-treated*) underwent a treatment designed to degrade DNA from the ends. Depleted windows (log_2_ ratio ≤ -1) were quantified by examining the first and last 2,500 bases of each chromosome, counting the number of depleted windows, summing across all chromosomes, and normalizing by the total number of windows in each sample to obtain the percentage. Each pair of bar plots represents results for the two samples using different combinations of *k*-mer length (*k*) and window size (*w*). As expected, across all scenarios, depletion was more pronounced in b06 (*high-treated* sample), reflecting greater end degradation with higher enzyme concentration.

Figure 4 demonstrates that the percentage of depleted windows across the entire genome was comparable between the two treated samples. Although both treated samples were derived from the same source as the untreated control, a small fraction of depleted windows appeared throughout the genome, likely due to the low sequencing coverage. However, as illustrated in Figure 5, when restricting the analysis to the first and last 2500 bases of each chromosome, we observed an overall increase in the proportion of depleted windows. Notably, depletion was more pronounced in sample b06 (*high-treated*), aligning with our expectations based on enzyme concentration.

These findings confirm that kdiff produces biologically meaningful results, successfully identifying the expected depletion patterns even in low-coverage and noisy nanopore sequencing data. Importantly, this was achieved without requiring any modifications to adapt the method for long-read sequencing.

## Discussion

In this study, we explored the suitability and potential of kdiff, a novel alignment-free method for detecting differences between sequencing datasets by analyzing differential *k*-mer abundances. By avoiding reliance on read mapping, kdiff effectively mitigates mapping biases and inaccuracies in reference genomes, providing a robust alternative for comparative genomic analyses. Additionally, kdiff significantly enhances computational efficiency compared to traditional mapping-based methods, making it particularly suitable for large-scale sequencing data analysis.

We validated kdiff in two biologically relevant applications. First, we applied it to the detection of genomic differences between normal and cancerous human samples. Our results showed strong agreement with those obtained using CNVkit, a standard mapping-based tool, while achieving significantly faster processing times. Furthermore, kdiff demonstrated consistency across different human genome assemblies, yielding stable results regardless of the reference used.

Second, we demonstrated effectiveness of our method to identify DNA depletion in complex telomeric regions using low-coverage nanopore sequencing data from yeast *Lodderomyces elongisporus*. Since no reference genome was available for the specific strain, an assembly from a related strain was used instead, further demonstrating robustness of kdiff with respect to the assembly used for the analysis.

In conclusion, kdiff produced consistent and meaningful results, highlighting its applicability to challenging genomic contexts, including the study of non-model organisms and suggesting its potential applicability to poorly assembled or fragmented genomes.

### Limitations of the study

Results of kdiff are influenced by two parameters: window size and *k*-mer length. In general, the choice of the window size affects the sensitivity and specificity of detection. Smaller windows are more sensitive to detecting shorter events but are also more susceptible to noise. Larger windows provide more stable signal but may miss smaller alterations. Similarly, in case of *k*-mer lengths, longer *k*-mers are more affected by sequencing errors and short polymorphisms, while short *k*-mers are less informative since they are not unique within the genome. We investigated the effect of both window size and *k*-mer length on *Lodderomyces elongisporus* data. Specifically, we tested window sizes of 100, 300, 500, and 1000 base pairs, combined with *k*-mers of length 15 and 21. Across all these configurations, kdiff reliably detected telomeric depletions. Nonetheless, we are unable to provide a more general guidance for choosing these parameters for specific use cases.

Another limitation is that kdiff cannot detect insertions of novel sequences that are not present in the reference, such as transposable elements and viruses. While it is technically possible to extend kdiff to analyze *k*-mers absent from the reference genome, our method intentionally focuses only on *k*-mers present in the reference to ensure that detected variations can be directly interpreted in a broader genomic context.

Finally, kdiff assigns the same ratio to identical *k*-mers across the genome, effectively reporting the average copy number of a given sequence rather than resolving copy number differences within repeat regions. In contrast, mapping-based approaches can, in some cases, distinguish repeat copies thanks to small variations present in the reference. While the kdiff behavior is advantageous in case of lower genome quality, where such small variations are not reliably characterized, it can be limiting for high quality assemblies highly similar to the analyzed samples.

## Resource availability

### Lead contact

Requests for further information and resources should be directed to and will be fulfilled by the lead contact, Tomáš Vinař (tomas.vinar@fmph.uniba.sk).

### Materials availability

This study did not generate new materials.

### Data and code availability

• Original code has been deposited at Zenodo under the https://doi.org/10.5281/zenodo.17053100 and is publicly available.
• The *Lodderomyces elongisporus* sequencing data was deposited in the European Nucleotide Archive (ENA) under BioProject accession number ENA: PRJEB96892 (runs ENA: ERR15515232, ERR15515233, ERR15515237).
• Any additional information required to reanalyze the data reported in this paper is available from the lead contact upon request.

## Acknowledgments

This research was supported in part by the 10.13039/100009040Slovak grant agency VEGA grants 1/0538/22 (T.V.) and 1/0140/25 (B.B.) and by the 10.13039/100023551Slovak Research and Development Agency grant APVV-22-0144 (J.N.). The research was also supported by the 10.13039/100000065EU Horizon 2020 program under the 10.13039/100010665Marie Skłodowska-Curie grant agreements no. 872539 (PANGAIA) and no. 956229 (ALPACA) and by the 10.13039/100018693Horizon Europe programme grant agreement no. 101180581 (ASVA-CGR) to L.D.

## Author contributions

Conceptualization, T.V., B.B., and A.G.; methodology, A.P., L.D., A.G., J.N., B.B., and T.V.; software implementation, A.P., L.D., and A.G.; sequencing experiment, V.H. and J.N.; data analysis, A.P. and L.D.; writing – review and editing, A.P., J.N., B.B., and T.V.; funding acquisition, T.V., B.B., J.N., and L.D.; supervision T.V. and J.N.

## Declaration of interests

The authors declare no competing interests.

## Declaration of generative AI and AI-assisted technologies in the writing process

During the preparation of this work, the author(s) used ChatGPT in order to improve grammar and stylistics. After using ChatGPT, the authors reviewed and edited the content as needed and take full responsibility for the content of the publication.

## STAR★Methods

### Key resources table

**Table:** 

REAGENT or RESOURCE	SOURCE	IDENTIFIER
**Biological samples**		

Lodderomyces elongisporus	Westerdijk Fungal Biodiversity Institute	CBS 5301

**Chemicals, peptides, and recombinant proteins**		

Nuclease BAL-31	New England Biolabs	Cat# M0213

**Critical commercial assays**		

Genomic-tip 100/G	Qiagen	Cat# 10243
Rapid Barcoding kit	Oxford Nanopore Technologies	SQK-RBK004
MinION FlowCell	Oxford Nanopore Technologies	FLO-MIN106 (R9.4.1)

**Deposited data**		

L. elongisporus control sample without BAL-31 treatment (nanopore reads)	This study	ENA: ERR15515232
L. elongisporus sample treated with 1U of BAL-31 (nanopore reads)	This study	ENA: ERR15515233
L. elongisporus sample treated with 2U of BAL-31 (nanopore reads)	This study	ENA: ERR15515237
Human cancer Illumina reads	Fang et al.^30^	SRR7890905
Human healthy Illumina reads	Fang et al.^30^	SRR7890940
Human reference genome	UCSC Genome Browser	hg38
Human reference genome	UCSC Genome Browser	hg17
Human reference genome	UCSC Genome Browser	hs1
Lodderomyces elongisporus strain NRRL-YB4239	Hoyer et al. 2023^42^	GCF_030384665.1

**Software and algorithms**		

kdiff	This study	https://github.com/fmfi-compbio/kdiff
CNVkit	Talevich et al. 2016^6^	https://github.com/etal/cnvkit
liftOver	Hinrichs et al. 2006^36^	https://github.com/ucscGenomeBrowser/kent
Guppy v.4.4.1	Oxford Nanopore Technologies	https://nanoporetech.com/software/other/guppy

### Experimental model and study participant details

The yeast *Lodderomyces elongisporus* strain CBS 5301 was grown overnight in liquid YPD medium (1% (w/v) yeast extract, 2% (w/v) peptone, 2% (w/v) glucose) at 28 ^∘^ C with constant aeration. *L. elongisporus* is a diploid and homothallic yeast species, yet it does not contain the complete mating type loci and the nature of its sexual reproduction remains unknown.^43^ The yeast authentication was performed by whole genome sequencing and the sequence comparison to the reference genome of the *L. elongisporus* type strain CBS 2605/NRRL YB-4239.

### Method details

The kdiff method is designed to identify differential genomic regions between two samples by leveraging differential *k*-mer abundances. It requires as input a reference genome and two sets of sequencing reads to be compared. Instead of relying on mapping, kdiff partitions the reference genome into non-overlapping windows of user-defined length and determines the degree of enrichment or depletion of each window based on its *k*-mer abundance profiles.

#### Counts of *k*-mers and their normalization

In the first step, *k*-mer counts are computed for each sample of sequencing reads using KMC3 tool.^44^ To account for both strands, we use canonical *k*-mers. Namely, the canonical form of *k*-mer *x* is the lexicographically smaller sequence out of *x* and its reverse complement. Most experiments used *k* = 21.

Since the two read sets have different total sequencing depths, we normalize the counts using the average *k*-mer coverage of each sample. Let *X*_*i*_ and *Y*_*i*_ denote the number of occurrences of *k*-mer *i* in the first and second read sets, *X* and *Y*, respectively, and let *N* be the number of *k*-mer positions in the reference genome *G* (for a single chromosome we have *N* = |*G*|-*k*+1). The average *k*-mer coverages, *c*_*X*_ and *c*_*Y*_, are calculated as:

(Equation 1) $$c_{X}=\frac{\sum_{i}X_{i}}{N},c_{Y}=\frac{\sum_{i}Y_{i}}{N}\text{,}$$

where *i* ranges over all distinct *k*-mers found in the respective read set.

#### Abundance ratio

For each *k*-mer position *i* in reference genome *G* we compute its abundance ratio *r*_*i*_ as follows:

(Equation 2) $$r_{i}=\frac{\left(X_{i}+\varepsilon_{X}\right)/c_{X}}{\left(Y_{i}+\varepsilon_{Y}\right)/c_{Y}}\text{.}$$

Here *ε*_*X*_ and *ε*_*Y*_ are small pseudocounts introduced to prevent division by zero. Note that pseudocounts need to be proportional to the sequencing depth of a sample in order to obtain a ratio equal to 1 (corresponding to a neutral state) when a *k*-mer is present in the reference genome but absent in both analyzed samples. This scenario may arise, for instance, when both samples share the same single nucleotide polymorphism at a specific position on the reference genome. We set these pseudocounts as follows:

(Equation 3) $$\varepsilon_{X}=\varepsilon \cdot \frac{c_{X}}{c_{X}+c_{Y}},\varepsilon_{Y}=\varepsilon \cdot \frac{c_{Y}}{c_{X}+c_{Y}}\text{.}$$

Here, *ε* is set to 0.0001.

#### Scoring windows

For a given window *w* of reference genome *G* we compute the relative copy number *R*(*w*) as the median of abundance ratios *r*_*i*_ for all *k*-mer starting positions *i* located in the window. This ratio characterizes how much a genomic window is enriched or depleted in one sample relative to the other. For instance, in a diploid genome, where each sample is expected to have two copies of each chromosome, a value of *R*(*w*) = 0.5 can correspond to a situation where the control sample *Y* contains two copies of the window as expected, whereas one copy was lost in the treated sample *X*. Similarly, total loss of the window in *X* would lead to ratio of 0, gain of one copy to ratio of 1.5 and gain of two copies to ratio of 2.

#### Sequencing of *Lodderomyces elongisporus* genomic DNA

To analyze telomeric repeats of the yeast *Lodderomyces elongisporus* strain CBS 5301 using the BAL31-NGS protocol inspired by Peška et al.,^40^ we extracted high-molecular-weight genomic DNA (as in Brejová et al.^45^) and purified by anion-exchange chromatography on a Genomic-tip 100/G (Qiagen). DNA samples (∼5 *μg*) were then digested with 1 and 2 U of BAL-31 (New England Biolabs) in 50 *μL* of 1× BAL-31 reaction buffer at 30^∘^ C for 15 min. The sequencing library was prepared using a Rapid Barcoding kit (Oxford Nanopore Technologies SQK-RBK004), sequenced on a MinION MK-1b with an FLO-MIN106 (R9.4.1) flowcell and basecalled using Guppy v.4.4.1. This resulted in one control sample and two treated samples obtained with different enzyme concentrations. The median coverage was 17 for the control and 15 for the two treated samples.

### Quantification and statistical analysis

No statistical analysis was used to analyze data in this article.

### Additional resources

Software availability: https://github.com/fmfi-compbio/kdiff.
